# Obituary

**Published:** 2009-04

**Authors:** Ramesh K. Goyal

**Affiliations:** Vice Chancellor, The Maharaja Sayajirao University of Baroda Vadodara - 390 002, Gujarat, India. E-mail: goyalrk@rediffmail.com

**Figure:**
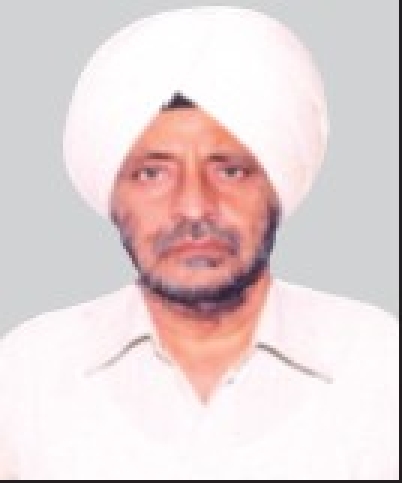
Prof. Manjeet Singh (1946-2009) Ex. Dean and Professor, Punjabi University, Patiala Ex. Professor, Department of Pharmacology, University Institute of Pharmaceutical Sciences The Immediate Past President of the Indian Pharmacological Society

Prof. Manjeet Singh, the immediate Past President and Executive Member of the Indian Pharmacological Society passed away on 30^th^ March 2009 after a brief illness.

Born in a small town of Punjab on 18^th^ April 1946, Dr. Singh obtained BPharm, MSc (Pharmacology) and PhD from Punjab University, Chandigarh. He started his career in 1973 in the Department of Pharmaceutical Sciences (now known as the University Institute of Pharmaceutical Sciences) at the University. He established a cardiovascular sciences laboratory in the department and was known for original basic research in ischemia reperfusion injury and oxidative stress. Dr. Singh joined Punjab University, Patiala in 1994, as Professor and Dean and established the Department of Pharmaceutical Sciences and Drug Research. During his stay, Dr Singh and his team of PhD students described the concept of resident mast cell in heart and its role in ischemia reperfusion injury. They also identified several other molecular targets for new drug development for cardio-protection. This research was very well recognized and a large number of awards were conferred on him such as: Fellow of International Academy of Cardiovascular Science, KG Nair Oration, Dr. OD Gulati Award, Achari Oration, PP Surya Kumari Award, Univas Prize, Achari Prize, NS Dhalla Oration and many more.

Prof Singh's latest achievement was the establishment of a model private college of pharmacy (Indo-Soviet Friendship College of Pharmacy) at Moga, Punjab. This college has a unique establishment with “state-of-the-art” research facilities not only in pharmacology but also other disciplines of pharmacy. Although this institute is in a small remote town in Punjab, it attracted a large number of experts as devoted faculty. In November 2008, a high profile scientific conference was also organized under his leadership.

Our Society has lost a dynamic and enthusiastic member; the pharmacy community has lost an affectionate and popular teacher, the pharmacology especially cardiovascular fraternity has lost a proud researcher and prolific writer, who made significant and original contributions in the field of basic cardiovascular physiology and pharmacology.

